# Editorial: Deep Learning in Aging Neuroscience

**DOI:** 10.3389/fninf.2020.573974

**Published:** 2020-10-26

**Authors:** Javier Ramírez, Juan M. Górriz, Andrés Ortiz, James H. Cole, Martin Dyrba

**Affiliations:** ^1^Department Signal Theory, Networking and Communications, University of Granada, Granada, Spain; ^2^Department of Communications Engineering, University of Málaga, Málaga, Spain; ^3^Department of Computer Science, Centre for Medical Image Computing, University College London, London, United Kingdom; ^4^Dementia Research Centre, Institute of Neurology, University College London, London, United Kingdom; ^5^German Center for Neurodegenerative Diseases (DZNE), Rostock, Germany

**Keywords:** deep learning, convolutional neural networks, brain age estimation, neurodegenerative diseases, automated diagnosis, brain image segmentation

## 1. Introduction

Deep learning (DL) has revolutionized the field of artificial intelligence by enabling computational models consisting of multiple processing layers to learn abstract representations of data (Hinton et al., 2006; Bengio et al., 2006). Conventional machine learning methods have been limited for decades by the need of expert knowledge to design sophisticated feature extraction algorithms in the process of transforming raw data into a suitable form for classification. In contrast, deep leaning methods, as representation-learning techniques, enable the learning model to be fed directly with raw data in order to discover the representations needed for classification (Krizhevsky et al., 2017; LeCun et al., 2015).

Currently, an intensive research effort is being devoted to the development of novel neuroimaging techniques to better understand the mechanisms of the central nervous system (CNS) and to early recognize age-related neural diseases (Payan and Montana, 2015; Sarraf and Tofighi, 2016; Martinez-Murcia et al., 2020; Martinez-Murcia et al., 2018, 2016) Ortiz et al.. The vast amount of data provided by large multicentre studies investigating new biomarkers for age-related neural diseases presents an opportunity for the development of more accurate deep learning models for early recognition of neurodegeneration as well as the characterization of the progressive course of neural disorders (Cole and Franke, 2017; Marzban et al., 2019; Segovia et al., 2018; Ortiz et al., 2016; Wang et al., 2018).

## 2. Research Topic Content

The aim of this research topic “Deep Learning in Aging Neuroscience,” published in *Frontiers in Aging Neuroscience* and *Frontiers in Neuroinformatics*, was to present the current state of the art in the theory and practice of deep learning computational modeling techniques in aging neuroscience with special emphasis on advancing our understanding of the mechanisms of CNS aging and age-related neural diseases. The research topic features 9 research articles. Most of the contributions examined disease progression and the relationships between different underlying pathological changes. Based on their contributions, the research articles were grouped into three main areas: brain age estimation (2 papers), automatic diagnosis of neurodegenerative diseases (5 papers), and brain image segmentation models (2 papers).

### 2.1. Brain Age Estimation

Predicting brain age based on structural and functional image data is still a challenging problem. Unveiling the normal aging of the brain is crucial in understanding the neural correlates of cognitive aging and neurodegenerative diseases such as Alzheimer's disease (AD).

Two papers of the research topic focused on brain age prediction by means of 18F-FDG brain metabolic topography data and structural T1-weighted MRI brain scans. Choi et al. proposed a deep learning model for predicting future brain metabolic topography by generating brain PET images. The generative model was based on a variational autoencoder (VAE). It used an 18F-FDG PET subject image and current age information as inputs to extract low-dimensional representation latent features that served as a basis for generation of PET image patterns corresponding to different brain ages. It was shown that, in spite of individual variability in age-related change, future regional metabolic changes were precisely predicted. The paper by Amoroso et al. presented an approach to predict brain aging based on structural T1-weighted MRI brain scans. They combined a complex network framework with deep learning strategies. Multiplex networks consisting of many layers were constructed, with each layer representing a single subject, the nodes being anatomical brain regions and the connections being derived from their pairwise similarities. A deep neural network processed nodal metrics, evaluating both the intensity and the uniformity of connections, to predict subjects' ages. The model yielded high accuracies and compared favorably with other state-of-the-art approaches.

### 2.2. Automatic Diagnosis of Neurodegenerative Diseases

This research topic also grouped different approaches for automatic diagnosis of neurodegenerative diseases based on deep learning classification models. Qiao et al. proposed a deep learning classification framework which performed multivariate data-driven feature extraction for automatic diagnosis of AD. The method was based on a three-level hierarchical partner matching independent component analysis (3LHPM-ICA) and Granger causality (GC) to infer the directional interaction between the independent components and to extract the effective connectivity features. Finally, a directed acyclic graph (DAG) neural network was used for classification. The proposed methodology was evaluated on a resting-state fMRI dataset consisting of 34 AD dementia patients and 34 normal controls (NCs) leading to a classification accuracy of 95.6%, with a sensitivity of 97.1% and a specificity of 94.1% with leave-one-out cross validation.

The paper by Qureshi et al. showed an evaluation of functional decline in AD dementia using three-dimensional convolutional neural networks (3D-CNN) and group ICA to model functional connectivity from resting-state fMRI data. The authors divided the dataset of AD patients into two groups based on dementia severity with respect to clinical dementia rating (CDR) scores: very mild to mild (CDR: 0.5–1) vs. moderate to severe (CDR: 2–3) dementia. Results reported a mean balanced classification accuracy of 92.3%, with specificity of 94.6% and sensitivity of 89.6%. In addition, medial frontal, sensorimotor, executive control, dorsal attention, and visual networks were found to be correlated with dementia severity.

Deep learning techniques showed improved classification performance in many medical imaging tasks including AD detection based on structural MRI data. However, these models are still perceived as being highly non-transparent and difficult to translate into clinical practice. The paper by Böhle et al. proposed layer-wise relevance propagation (LRP) to visualize CNN decisions for AD based on MRI data. This technique yields importance or relevance maps indicating how much each voxel is contributing to the final classification outcome, thus showing the potential of LRP to assist clinicians in interpreting neural network decisions.

The systematic review by Jo et al. presents recent studies using deep learning and neuroimaging data for diagnostic classification of AD. The authors included 16 research articles published between 2013 and 2018 and classified them according to deep learning algorithms and neuroimaging data types. Current state-of-the-art DL approaches yielded accuracies of up to 96.0% for AD dementia classification and 84.2% for the prediction of conversion from mild cognitive impairment (MCI) to dementia. The latter is of particular clinical relevance, as this could eventually lead to early identification of AD patients, enabling stratification for clinical trials and targeted interventions to delay dementia onset. However, the current accuracy of approximately 85% is likely too low for clinical adoption.

A deep learning approach for Parkinson's disease (PD) detection using isosurface-based features and convolutional neural networks was presented in Ortiz et al.. The authors proposed the use of isosurfaces as a solution to efficiently reduce the amount of data while keeping the most relevant information. Here, isosurfaces connect voxels above a specified intensity in a way similar to contour lines connecting points of equal elevation. These isosurfaces were then used to implement a classification system based on the CNN architectures LeNet and AlexNet. An average accuracy of 95.1% and AUC of 0.97 was achieved to differentiate PD patients and controls using 123I-Iofluopane (DaTSCAN) SPECT images. Finally, saliency maps of the last two-neuron layer were provided to determine which areas of the input brain images had a greater contribution to the predicted output class.

### 2.3. Brain Image Segmentation

Another important topic of research in the field of aging neuroscience is the development of image segmentation techniques for the assessment of disease progression. The paper by Jeong et al. focused on the segmentation of white matter hyperintensities (WMH) that appear as regions of abnormally high signal intensity on T2-weighted magnetic resonance image (MRI) sequences. This imaging marker has been identified as valuable for dementia and brain aging processes in age-related neuroscience. The authors developed and evaluated a saliency U-Net with irregularity age map (IAM) to decrease the U-Net architectural complexity without performance loss. Their so-called Dilated Saliency U-Net for WMH segmentation reduced the training complexity of the original U-Net segmentation network and improved the Dice coefficient score to 0.56 with a sensitivity of 47.5%.

Segmentation of ischaemic stroke lesions remains a challenge in neuroimaging, especially when dealing with magnetic resonance perfusion imaging data. Deep learning CNN architectures developed to date reported low performance when segmenting ischaemic stroke lesions due to the lesion heterogeneity with respect to location, shape, size, image intensity and texture. The paper by Pérez Malla showed an evaluation of enhanced learning techniques for segmenting ischaemic stroke lesions in brain magnetic resonance perfusion images using a CNN scheme. In this way, data augmentation, transfer learning and post-processing techniques were evaluated for the segmentation of stroke lesions using the ISLES 2017 dataset, which contains expert annotated diffusion-weighted perfusion and diffusion brain MRI of 43 stroke patients. Among the experiments conducted, data augmentation combined with a binary hole filling procedure achieved the best results, improving the mean Dice score by 17% compared to the baseline model.

## Author Contributions

All authors listed have made a substantial, direct and intellectual contribution to the work, and approved it for publication.

## Conflict of Interest

The authors declare that the research was conducted in the absence of any commercial or financial relationships that could be construed as a potential conflict of interest.

## References

[B1] Bengio Y. Lamblin P. Popovici D. Larochelle H. (2006). Greedy layer-wise training of deep networks. In Proceedings of the 19th International Conference on Neural Information Processing Systems, NIPS - 06 (Cambridge, MA: MIT Press), 153–160.

[B2] Cole J. H. Franke K. (2017). Predicting age using neuroimaging: innovative brain ageing biomarkers. Trends Neurosci. 40, 681–690. 10.1016/j.tins.2017.10.001 29074032

[B3] Hinton G. E. Osindero S. Teh Y.-W. (2006). A fast learning algorithm for deep belief nets. Neural Comput. 18, 1527–1554. 10.1162/neco.2006.18.7.1527 16764513

[B4] Krizhevsky A. Sutskever I. Hinton G. E. (2017). ImageNet classification with deep convolutional neural networks. Commun. ACM 60, 84–90. 10.1145/3065386

[B5] LeCun Y. Bengio Y. Hinton G. (2015). Deep learning. Nature 521, 436–444. 10.1038/nature14539 26017442

[B6] Martinez-Murcia F. J. Górriz J. M. Ramírez J. Ortiz A. (2016). A structural parametrization of the brain using hidden Markov models-based paths in Alzheimer's disease. Int. J. Neural Syst. 26:1650024. 10.1142/S0129065716500246 27354189

[B7] Martinez-Murcia F. J. Górriz J. M. Ramírez J. Ortiz A. (2018). Convolutional neural networks for neuroimaging in Parkinson's disease: is preprocessing needed? Int. J. Neural Syst. 28:1850035. 10.1142/S0129065718500351 30215285

[B8] Martinez-Murcia F. J. Ortiz A. Gorriz J. Ramirez J. Castillo-Barnes D. (2020). Studying the manifold structure of Alzheimer's disease: a deep learning approach using convolutional autoencoders. IEEE J. Biomed. Health Inform. 24, 17–26. 10.1109/JBHI.2019.2914970 31217131

[B9] Marzban E. N. Teipel S. J. Buerger K. Fliessbach K. Heneka M. T. Kilimann I. (2019). P3-361: explainable convolutional networks and multimodal imaging data: the next step towards using artificial intelligence as diagnostic tool for early detection of Alzheimer's disease. Alzheimers Dement. 15(7S_Part_21), P1083–P1084. 10.1016/j.jalz.2019.06.3394

[B10] Ortiz A. Munilla J. Górriz J. M. Ramírez J. (2016). Ensembles of deep learning architectures for the early diagnosis of the Alzheimer's disease. Int. J. Neural Syst. 26:1650025. 10.1142/S0129065716500258 27478060

[B11] Payan A. Montana G. (2015). Predicting Alzheimer's disease: a neuroimaging study with 3D convolutional neural networks. arXiv preprint arXiv:1502.02506.

[B12] Sarraf S. Tofighi G. (2016). DeepAD: Alzheimer's Disease Classification via Deep Convolutional Neural Networks using MRI and fMRI. Cold Spring Harbor Laboratory. Available online at: https://www.biorxiv.org/content/early/2016/08/21/070441

[B13] Segovia F. Górriz J. M. Ramírez J. Martinez-Murcia F. J. García-Pérez M. (2018). Using deep neural networks along with dimensionality reduction techniques to assist the diagnosis of neurodegenerative disorders. Logic J. IGPL 26, 618–628. 10.1093/jigpal/jzy026 30532642 PMC6267552

[B14] Wang S.-H. Phillips P. Sui Y. Liu B. Yang M. Cheng H. (2018). Classification of Alzheimer's disease based on eight-layer convolutional neural network with leaky rectified linear unit and max pooling. J. Med. Syst. 42:85. 10.1007/s10916-018-0932-7 29577169

